# Case report: long-term clinical remission of food-responsive enteropathy in a dog fed a yellow mealworm-based diet

**DOI:** 10.3389/fvets.2026.1796650

**Published:** 2026-10-13

**Authors:** Remigiusz Gałęcki, Ismena Gałęcka

**Affiliations:** 1 Department of Veterinary Prevention and Feed Hygiene, Faculty of Veterinary Medicine, University of Warmia and Mazury in Olsztyn, Olsztyn, Poland; 2 Department of Epizootiology, Faculty of Veterinary Medicine, University of Warmia and Mazury in Olsztyn, Olsztyn, Poland

**Keywords:** chronic inflammatory enteropathies, edible insects, FRE, gall bladder, hypoallergenic, novel protein, sensitive diet, *Tenebrio molitor*

## Abstract

Food-responsive enteropathy (FRE) is the most common form of canine chronic inflammatory enteropathy. Novel protein diets constitute first-line therapy, and many dogs have already been exposed to “novel” meat alternatives. Edible insects are a novel, highly digestible, and sustainable protein source. In the present study, a five-year-old male York-shire Terrier with chronic FRE and gallbladder sludge was transitioned to a diet with a 35% inclusion level of *Tenebrio molitor* larvae meal. Clinical indices improved from “severe” (CIBDAI = 9 and CCECAI = 11) to “clinically insignificant” (CIBDAI = 0 and CCECAI = 2) within 3 weeks and remained stable for 24 months. Body weight normalized (from 5.4 to 4.0 kg), coat quality improved, and the ultrasound examination revealed that gallbladder sludge was eliminated. A brief interruption of the insect-based diet coincided with the recurrence of sludge and gastrointestinal signs, both of which resolved after the reintroduction of *T. molitor* larvae meal. Hematological and serum biochemical parameters remained within the reference ranges. The mealworm-based diet led to long-term remission of FRE and elimination of gallbladder sludge without adverse effects. Insect protein is a viable hypoallergenic option for canine elimination diets and may convey additional gut and hepatobiliary benefits.

## Introduction

Canine chronic inflammatory enteropathies (CIE) are a group of disorders characterized by persistent or recurrent diarrhea, vomiting, anorexia, weight loss, abdominal pain that have no identifiable extraintestinal cause (1, 2). Of these, food-responsive enteropathy (FRE) is the most common subtype that accounts for up to 50–70% of chronic enteropathies in dogs (1, 2). The affected dogs usually improve within 2–4 weeks when placed on an elimination diet containing a novel protein or a hydrolyzed protein source (3, 4). The selection of novel, less antigenic protein sources is critical, as many dogs have been exposed to common dietary ingredients (5, 6). Response to a specific dietary intervention can vary significantly depending on the study population, diet type, number of dietary trials, follow-up period, and criteria used to define response (7, 8).

Therefore, the impact of a diet should be interpreted cautiously (9) and in the context of multifaceted clinical management, particularly in patients requiring supportive care during disease exacerbation or stabilization. These findings have increased the interest in alternative proteins for hypoallergenic diets.

Edible insects have emerged as a promising novel protein source in pet nutrition, because they combine nutritional value with potential environmental advantages (3, 6). Insects provide proteins of high nutritional value, contain essential amino acids and lipids, and when included in a complete dog diet, can be easily digestible (5, 6). The larvae of the yellow mealworm (*Tenebrio molitor*) are among the leading candidates for pet nutrition (6, 10–12). According to the literature, the quality and nutritional content of *T. molitor* protein is comparable to that of animal-derived products (13, 14). Subsequent research has confirmed that the apparent total tract digestibility of insect-based diets is comparable to that of traditional diets (15). From a clinical perspective, insect protein may be particularly useful in dogs with chronic gastrointestinal symptoms and suspected FRE, especially when previous exposure to common or unconventional animal proteins limits the availability of truly novel dietary options. Case studies have shown that BSF-based diets were well tolerated by dogs with severe food allergies and led to clinical improvement (5). Therefore, *T. molitor*-based diets may also be a realistic original (alternative) protein supply strategy for selected patients requiring an elimination or hypoallergenic diet. However, a cautious approach is still needed because insect proteins share some epitopes with other allergens. In example, Premrov et al. demonstrated that dogs allergic to dust mites can exhibit IgE cross-reactivity to mealworm proteins (16).

The aim of this case study is to analyze the impact of a mealworm-based diet in a dog with FRE. To the best of the authors' knowledge, this is the first longitudinal study to report on the resolution of chronic enteropathy as well as a concomitant improvement in gallbladder pathology (biliary sludge) in a dog fed an insect-based diet.

## Case description

The described case concerns a 5-year-old male Yorkshire Terrier. This breed have been reported to be associated with complex chronic enteropathies, including Yorkshire terrier-associated enteropathy and food responsive protein-losing enteropathy (FR-PLE) (17, 18). Therefore, long-term dietary management of chronic gastrointestinal symptoms in this breed may be of particular clinical interest. The owner reported gastrointestinal problems, namely recurrent vomiting, diarrhea, and moderate abdominal pain. The dog remained under the care of a veterinarian, who introduced the following treatment. Body weight ranged from 4.5 to 5.4 kg [body condition score (BCS) 6 or 7/9, i.e., overweight]. The treatment included dietary changes (introducing a new protein source and a hydrolyzed diets based on, among others, chicken, pork, soy protein), routine antiparasitic prophylaxis (combination preparations such as milbemycin oxime and praziquantel or fenbendazole and praziquantel every 3 months according to the recommendations of the European Scientific Counsel Companion Animal Parasites; regular prophylaxis against ectoparasites was also carried out using fluralaner, also every 3 months), antibiotic therapy, and anti-inflammatory therapy. Initially, only dietary modifications brought temporary clinical improvement, which worsened again after a few days; the dog also often lost interest in the new diet. Due to right and left atrioventricular valve insufficiency and degenerative atrioventricular valve disease stage C (ACVIM), the owner did not consent to a diagnostic tests requiring anesthesia. Cardiac treatment included pimobendan, furosemide, and benazepril. The patient was also diagnosed with tracheal collapse, which was treated with glucosamine and chondroitin supplements. In January 2021 ultrasound revealed biliary sludge, without concomitant thickening of the gallbladder wall and without dilatation or obstruction of the bile ducts. Ursodeoxycholic acid was administered. Previous treatment may have been compromised by the use of several interventions, including dietary changes, antibiotics, anti-inflammatory therapy, and gastrointestinal medications. However, these measures provided only temporary or incomplete improvement and did not lead to lasting remission. Current antimicrobial care recommendations are against routine antibiotic use in dogs with diarrhea unless severe systemic disease, bacterial infection, or sepsis is suspected.

One month later (in February 2021), the owner decided to change the treatment facility and the dog was admitted to the authors' university veterinary clinic, where he was managed under the veterinary supervision of the authors. Because CIE was suspected, both the CIBDAI and the CCECAI were assessed; the results are presented in Table 1 (CIBDAI = 9, CCECAI = 11), which is consistent with severe disease activity and clinically significant disease. The dog was placed on a dry lamb diet and a wet venision diet used as a medication carrier (administering oral medications alone exacerbated vomiting). Ultrasound examination confirmed changes in the gallbladder, but no changes in other organs were noted (including bowel wall thickening or changes in echogenicity) (19). Pancreatitis was effectively ruled out by a test results (i.e. normal canine pancreatic lipase immunoreactivity (cPLI <30 μg/L), therapies conducted and treatment effects. Cholecystitis was considered but deemed unlikely given the absence of hallmark signs (no abdominal pain after eating, jaundice, or fever) and unremarkable hepatobiliary findings (normal liver enzymes and total bilirubin), making active gallbladder inflammation very unlikely. Likewise, hypoadrenocorticism was excluded based on normal serum electrolyte levels (no hyponatremia or hyperkalemia), cortisol level 146,23 nmol/L (range 24.83–124.16nmol/L), and the presence of an appropriate stress leukogram. Additionally, the dog's normal serum albumin and glucose concentrations further reduce suspicion of hypoadrenocorticism.

**Table 1:** Clinical severity evaluation based on the Canine IBD Activity Index (CIBDAI) and the Canine Chronic Enteropathy Clinical Activity Index (CCECAI) exact time points (baseline, 3 weeks, 12 months, 24 months, post-poisoning, and final remission) the dietary intervention (mealworm-based diet vs. other foods).

Variables		Other foods (baseline)		*Tenebrio molitor*-based diet (3 weeks)		*Tenebrio molitor*-based diet (12 months)		*Tenebrio molitor*-based diet (24 months)		Other foods (post-poisoning)		*Tenebrio molitor*-based diet (final remission)	
CIBDAI	CCECAI	CIBDAI	CCECAI	CIBDAI	CCECAI	CIBDAI	CCECAI	CIBDAI	CCECAI	CIBDAI	CCECAI	CIBDAI	CCECAI
Attitude/ activity		0		0		0		0		0		0	
Appetite		2		0		0		0		2		0	
Vomiting		3		0		0		0		2		0	
Stool consistency		2		0		0		0		1		0	
Stool frequency		2		0		0		0		1		0	
Weight loss		0		2^*^		0		0		0		0	
–	Albumin levels	–	0	–	0	–	0	–	0	–	0	–	0
–	Ascites and peripheral edema	–	0	–	0	–	0	–	0	–	0	–	0
–	Pruritus	–	2	–	0	–	0	–	0	–	1	–	0
**Total**		9 (severe disease activity)	11 (severe disease activity)	2 (clinically insignificant)	2 (clinically insignificant)	0 (clinically insignificant)	0 (clinically insignificant)	0 (clinically insignificant)	0 (clinically insignificant)	6 (moderate disease activity)	7 (moderate disease activity)	0 (clinically insignificant)	0 (clinically insignificant)

^*^the dog scored 2 points after switching to the new diet due to weight loss (from 5.4 to 4.0kg). Importantly, the goal of the dietary intervention was to achieve a healthy weight, rather than cachexia.

Clinical signs (vomiting, diarrhea, belching, abdominal discomfort, bloating, and loose stools) were least severe with the lamb/venision diet. Exacerbations requiring veterinary intervention occurred approximately once a month. Treatment typically included subcutaneous or intravenous fluids, antiemetic therapy (maropitant), gastroprotective and antispasmodic medications (e.g., metamizole with hyoscine or buprenorphine for visceral pain), antidiarrheal medications, and probiotics containing *Lactobacillus plantarum* and *Bifidobacterium animalis*. These therapies resulted in improvement after 4–10 days. The administration of drugs was intended to be symptomatic treatment, not causal treatment. Due to clinical picture, the patient was considered a reasonable candidate for dietary management as the main long-term intervention.

The dog was switched to an experimental, non-commercial dry food developed specifically within the R&D project. The diet contained a 35% inclusion level of *T. molitor* larvae meal as the sole source of animal protein (25.83% of protein, 13.79% of fat, 5.37% of ash, 5.22% of fiber in a dry matter; gross energy 453.14 kcal/100 g, metabolizable energy 357.39 kcal/100 g). Its formulation and selected physicochemical properties have been described previously (20). No other changes in the dog's lifestyle were made at the time of the dietary transition. The new diet was introduced gradually over a period of around seven days, and it was well accepted by the dog. According to the owner, the dog eagerly ate insect-based granules and even showed a preference for the new diet over his previous food. A notable improvement in GI signs was observed two to three weeks after the dietary change: the dog's stools were well-formed and regular; vomiting ceased, and abdominal discomfort and bloating resolved. The dog's overall condition, energy levels, and appetite improved. In the following months, the dog's body weight decreased to around 4.0 kg (BCS 4–5/9, an ideal score) from, presumably due to the combined effects of the new diet and controlled food intake. After several months of adherence to the *T. molitor* diet, a follow-up ultrasound exam revealed a normal gallbladder with no visible sludge. The *T. molitor*-based diet was continued.

Due to the improvement in the condition, a diagnosis of FRE was suspected. In this case, the dog's clinical condition was relatively stable and showed a prompt, robust response to dietary therapy, which strongly pointed toward a food-responsive enteropathy. Furthermore, the diagnosis of FRE in presented case was confirmed independently by two board-certified gastroenterology specialists who reviewed the patient's history, response to therapy, and follow-up.

The dog was subjected to periodic recheck examinations involving blood tests and interviews with the owner. Reanalysis of the CIBDAI and CCECAI showed a significant improvement–two points on each scale, corresponding to clinically insignificant disease. In particular, chronic vomiting was eliminated, stool consistency was normalized, and appetite improved from moderate reduction to normal. Mild pruritus was also resolved. Weight loss was the only score component that was not fully normalized (the dog scored 2 points after switching to the new diet), but the goal of the dietary intervention was to achieve a healthy weight, rather than cachexia. CIBDAI and CCECAI scores are presented in Table 1.

Blood morphology and serum biochemistry panels were performed roughly every 6–12 months to monitor systemic health and screen for any issues related to the new diet. These results indicate that the insect-based diet provided adequate nutrition and did not cause any metabolic disturbances. Data are presented in Tables 2, 3.

**Table 2:** The results of hematology tests.

Parameter (reference range)	02/07/2022	19/09/2022	09/05/2023	02/08/2024	19/09/2024
RBC (5.65–8.87 M/μl)	7.37	7.47	6.72	7.62	6.66
HCT (37.3–61.7%)	50.5	51.8	45.5	50.4	39.1
HGB (13.1–20.5 g/dl)	17.2	17.6	15.5	18.7	10.5
MCV (61.6–73.5 fL)	68.5	69.3	67.7	66.3	48.2
MCH (21.2–25.9 pg)	23.3	23.6	23.1	24.6	15.8
MCHC (32–37.9 g/dl)	34.1	34	34.1	37.1	32.7
RDW (13.6–21.7%)	16.4	18.9	15.8	17.2	26.2
RETIC (10–110 K/μl)	54	98	24	106	30
WBC (5.05–16.76 K/μl)	15.8	14.09	11.29	23.11	12.13
NEU (2.95–11.64 K/μl)	8.82	9.55	17.51	16.8	7.15
LYM (1.05–5.1 K/μl)	5.04	5.88	2.8	4.16	3.62
EOS (0.06–1.23 K/μl)	0.95	0.85	0.19	0.76	0.22
BASO (0–0.1 K/μl)	0.05	0.03	0.1	0.02	0.03
PLT (148–484 K/μl)	172	222	204	201	344
MPV (8.7–13.2 fL)	11.3	11.4	10.9	11.2	10.3
PCT (0.14–0.16%)	0.19	0.25	0.22	0.23	0.31

**Table 3:** The results of blood biochemistry tests.

Parameter (reference range)	26/11/2021	02/07/2022	19/09/2022	09/05/2023	01/08/2024
ALB (2.3–4 g/dl)	3.3	3.5	3.4	3.6	4.6
ALKP (23–212 U/L)	63	97	129	54	130
ALT (10–125 U/L)	34	23	35	51	56
AMY (500–1,500 U/L)	826	847	735	528	864
AST (0–50 U/L)	31	26	28	27	26
BUN (7–27 mg/dl)	19	21	19	16	26
Ca (7.9–12 mg/dl)	10.8	10.2	10.5	10.2	11.5
CHOL (110–320 mg/dl)	123	214	203	174	176
CREA (0.5–1.8 mg/dl)	0.8	0.7	0.8	0.5	0.8
GGT (0–11 U/l)	2	4	3	2	4
GLU (74–143 mg/dl)	122	100	103	123	97
K (3.5–5.8 mmol/l)	3.9	4.2	4.8	3.7	4.2
LIPA (200–1,800 U/l)	1,084	1,664	1,614	1,331	1,484
Na (144–160 mmol/l)	135	154	154	154	155
PHOS (2.5–6.8 mg/dl)	4.1	5.3	4.5	3.8	5.6
TBIL (0–0.9 mg/dl)	<0.1	0.2	<0.1	0.3	0.4
TP (5.2–8.2 g/dl)	6.5	7	6.4	6.6	8
Na/K	35	37	32	42	37
GLOB (2.5–4.5 g/dl)	3.2	3.5	3	3	3.5
ALB/GLOB	1	1	1.1	1.2	1.3
TT4 (1–4 μg/dl)	1.2	3.1	1.6	2.3	2.4

For 2 years, the dog was fed *T. molitor* dog food. After this period, an episode of poisoning occurred (suspected toxicosis secondary to ingestion of unidentified material (garbage/human food) during an urban walk near a construction site, as reported by the owner), which led to moderate disease activity. The patient presented with vomiting, anorexia, and lethargy, and the results of diagnostic tests were indicative of acute gastritis, enteritis, pancreatitis, and cholecystitis). The dog was admitted to an intensive care unit and received IV fluids, antibiotics, analgesics, and antiemetics. Due to pancreatitis (Canine Specific Pancreatic Lipase 743 μg/L) it was necessary to introduce a reduced-fat diet.

The patient recovered from the acute episode within one to two weeks and was discharged. However, due to lingering anorexia and taste aversion after the illness, the dog refused to eat the low-fat prescription diet at home. The owner attempted to find alternative commercial low fat diets with <8% fat content. The challenge was finding a food with a protein source the dog hadn't been getting before, and with reduced fat. Ultimately, the dog was switched back to a lamb/venision-based diet. However, the owner reported a persistent reluctance to eat.

A recheck abdominal ultrasound was performed approximately 3 week after change of diet. Gallbladder sludge reappeared after nearly 2 years on the *T. molitor* diet. The above could be attributed to multiple factors, with the recent anorexia, rapid dietary changes as potential contributors and cholecystitis. The prescription GI diet, while low in fat, was moderate in fiber and contained ingredients that might have affected bile composition or gallbladder motility. Ursodeoxycholic acid was re-started to help dissolve the sludge. However, the treatment was discontinued by the owner due to difficulties with oral administration after 2 weeks.

Appetite issues persisted in the following 3 weeks. In total, the dog remained on low-fat food for 5 weeks. The owner decided to switch back to the *T. molitor*-based diet (given the dog's previous positive response and willingness to eat it), despite the higher amount of fat in dry matter than is recommended for acute pancreatitis. The dog's food intake improved immediately. One month after returning to *T. molitor*-based food, a follow-up ultrasound revealed that the sludge had once again cleared (anechoic, normal bile). This timeline suggests that the *T. molitor* diet might have played a role in maintaining gallbladder health because sludge appeared during the dietary break and was eliminated once the dog had resumed the diet. The patient remained on the *T. molitor*-based diet, and its condition was assessed as stable. Ultrasound images are presented in Figure 1.

**Figure 1:**
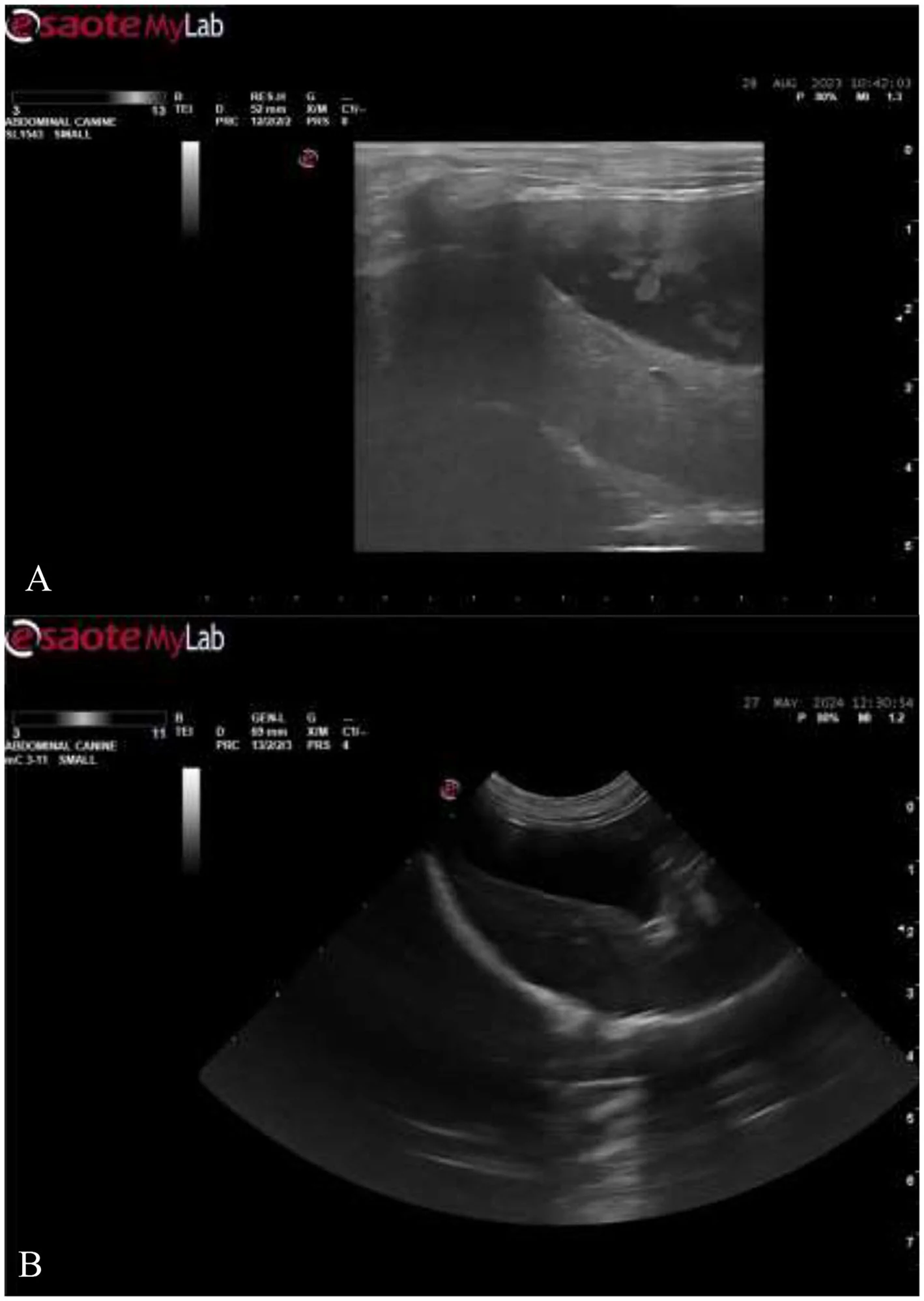
Comparative ultrasound images of the gallbladder. **(A)** echogenic bile with sludge accumulation (after the poisoning incident, the dog was fed low-fat food). At this stage, the dog was fed a low-fat diet, and when sludge was detected, ursodeoxycholic acid was resumed. Ursodeoxycholic acid was administered for approximately 2 weeks and then discontinued due to difficulty with oral administration.; **(B)** anechoic (black) gallbladder lumen and sludge clearing (9 months after reintroducing *T. molitor*-based feed). Ursodeoxycholic acid was no longer administered. The correlation between diet and gallbladder appearance as well as clinical data suggest that the mealworm-based diet not only helped manage the dog's intestinal disease, but may have also contributed to improved biliary function. However, the previous short-term use of ursodeoxycholic acid remains a limitation and prevents definitive attribution of the gallbladder improvement to diet alone.

The owner reported that dog's overall condition had shown improvement. FRE was effectively in remission as long as the dog stayed on the insect-based diet. There were no reports of exacerbation or recurrence of acute pancreatitis. Stools remained consistently well-formed, and defecation frequency normalized to 1–2 times a day. Throughout the described period, antiparasitic prophylaxis protocol was conducted in accordance with the patient's previous treatment history. The dog's coat, previously dull and prone to thinning, developed a healthy shine and fullness. No dermatological problems or pruritus were observed while on the insect-based diet. By the time this article was submitted for publication, the dog's FRE was well-controlled with diet alone. During the 24-month follow-up period, the patient's cardiac disease and tracheal collapse were clinically monitored during periodic veterinary check-ups including echocardiography every 6 months. The cardiac treatment regimen, remained stable during the dietary follow-up period. No changes in cardiac treatment were temporally associated with improvement in gastrointestinal symptoms or gallbladder findings. The dog did not require any other medications while on the *T. molitor* diet. Probiotics and other GI drugs were tapered off.

## Discussion

This case study demonstrated that an insect-based diet (*T. molitor* larvae) may be successfully used as a novel protein source to manage chronic FRE in a dog. Over studied period, the dog's GI signs improved to a point where clinical disease activity indices were stabilized within the normal range of values indicative of remission. A feature that diverged from the typical clinical picture in our case was the patient's elevated body condition score (BCS); however, this can occur when malabsorption is absent. In a study of dogs with chronic inflammatory enteropathy (CIE), Hodel et al. reported that 17% were overweight (BCS 6–7/9) (21). Another possible explanation in this case is potential colonic involvement in the pathogenesis and/or the owner's feeding practices. the dog's BCS was re-assessed during treatment and remained stable or improved once on the proper diet, further indicating that the diet was meeting his caloric needs and that the enteropathy was well-managed.

Gallbladder issues were also resolved after the patient had been placed on an insect-based diet. In dogs, biliary sludge is often an incidental finding that is not always clinically significant (22–24), but in the current study, the presence of sludge correlated with periods of GI problems and vomiting, which suggests it did have clinical relevance. Before the dietary change, the dog frequently vomited bile and had abdominal pain, and these signs can be associated with gallbladder dysmotility or cholestasis. After sustained feeding with the *T. molitor* diet, those signs abated, and ultrasound exams revealed an empty, normally functioning gallbladder. When the diet was briefly discontinued (due to the poisoning incident), sludge reappeared and vomiting recurred, and both issues once again resolved when the insect diet was resumed. This temporal association suggests a cause-and-effect relationship.

It is hypothesized that the low fat content and the fatty acid composition of the *T. molitor* diet could potentially have contributed to gallbladder emptying in this case. High-fat, high-cholesterol diets and obesity are known risk factors for bile stasis and sludge formation in dogs (24). In contrast, diets with a moderate fat content and balanced fatty acid composition may promote gallbladder contraction and bile flow. For example, omega-3 polyunsaturated fatty acids (PUFAs) have been reported to enhance gallbladder motility and alter prostaglandin synthesis in ways that could reduce biliary sludge (24). In the described case, the insect-based diet had an omega-6:omega-3 ratio of approximately 7.3:1. A very high omega-6:3 ratio can have pro-inflammatory effects, whereas a very low ratio can lead to oxidative stress or coagulopathies (25). A 7:1 ratio is moderate, and it might have contributed to an anti-inflammatory effect supporting biliary health. Additionally, *T. molitor* fat contains some lauric acid (C12:0) and other medium-chain triglycerides with antimicrobial and emulsifying properties (26, 27). Lauric acid can inhibit the growth of certain biliary pathogens and reduce bile sludge formation through its cleansing effects (28). These characteristics could theoretically influence bile composition or viscosity in presented case. However, bile acid composition, bile viscosity, and gallbladder ejection fraction were not measured. Therefore, this proposed mechanism remains speculative and should be interpreted cautiously as a hypothesis proposed from a single case observation.

*Tenebrio molitor* proteins have been shown to suppress pro-inflammatory cytokines in experimental models. For example, Shang et al. demonstrated that *T. molitor* peptide supplementation reduced the expression of TNF-α, IL-1β, and IL-6 genes in mice with intestinal injury (29). Similarly, Anusha and Negi reported significant reductions in the circulating levels of TNF-α, IL-6, and IL-1β in aged mice fed mealworm protein, as well as an increase in the levels of the IL-4 anti-inflammatory cytokine (30). These cytokines (TNF, IL-1, IL-6) are the key mediators in canine inflammatory bowel disease, contributing to mucosal inflammation. By lowering their levels, the insect diet may have helped restore normal intestinal immune homeostasis in the dog. These findings align with the observations made in the present study: the dog's GI problems resolved on the insect-based diet, hinting at reduced inflammatory activity. The CIBDAI score for vomiting decreased from 3 (severe) to 0, and stool consistency normalized, which could point to healing of the previously inflamed gut lining. Intestinal biopsies or cytokine assays were not performed in this case, but the clinical remission was consistent with what one might expect if intestinal inflammation were markedly reduced.

From a broader perspective, this case study adds to the growing body of evidence that insect-based pet foods provide functional benefits in managing some health conditions including FRE. In this scenario, insects offered a truly novel alternative. In the present study, novel terrestrial proteins (lamb and venision) did not lead to full recovery, and the patient achieved remission only after switching to an insect-based diet. The above implies that even unconventional protein sources can sometimes share cross-reactive epitopes, whereas insects are evolutionarily very distant from traditional livestock and thus less cross-reactive. Finally, it is worth noting that insect-based diets should be introduced with veterinary guidance, particularly in dogs with a complex medical history (6). It is important to emphasize that dietary therapy is most appropriate for dogs in a stable clinical state, with preserved physical condition, limited laboratory abnormalities, and a low suspicion of severe systemic disease. In patients with severe clinical manifestation, dietary intervention remains important, but may not be sufficient as a only therapeutic approach.

This case study has a number of potential limitations. Observations were made on a single animal without a controlled rechallenge (except for the dietary break caused by poisoning). The dog was administered the same cardiac medications and received the same level of care during the entire study; therefore, these factors are unlikely to explain the GI remission. The brief use of ursodeoxycholic acid somewhat confounds the gallbladder outcome. The proposed influence of the *T. molitor*-based diet on gallbladder function remains speculative. However, the fact that the sludge cleared only after switching to the insect-based diet despite recent ursodeoxycholic acid treatment (and was cleared once again by the diet) suggests that diet was the main factor in this case.

Due to the lack of consent from the owner, the histologic resolution of enteropathy was not confirmed by endoscopy and biopsy, and the assessment was primarily clinical. In practice, FRE is often managed based solely on the patient's clinical response to the diet, without the use of invasive diagnostics, especially when the patient is responding to the change of diet (9, 31, 32). Although a large-intestinal component of this dog's gastrointestinal disease cannot be ruled out, precise localization of the underlying pathology was not possible due to the owner's lack of consent for advanced diagnostic procedures.

The present case study of a canine patient has the potential to provide practical insights on how a *T. molitor*-based diet can be used to manage chronic enteropathy, achieve remission of GI symptoms, and provide ancillary benefits to skin and gallbladder health. These findings may support the view that insects are not only an environmentally-sustainable protein source, but also a clinically effective solution for patients with FRE. The observed positive outcomes, including high palatability, nutritional adequacy, resolution of clinical signs, and no adverse effects, indicate that insect-based diets might have the potential to be integrated into mainstream veterinary dietary therapy.

## Data Availability

The original contributions presented in the study are included in the article/supplementary material, further inquiries can be directed to the corresponding author.
